# Tuberculosis detection actions in Primary Health Care during the COVID-19 pandemic

**DOI:** 10.1590/0034-7167-2024-0389

**Published:** 2025-12-08

**Authors:** Isabella Carlini Bulizani, Stephanie Ribeiro, Ana Carolina Silva, Thais Tiemi Yamamoto, Rachel Russo Leite, Hugo Fernandes, Roxana Isabel Cardozo Gonzales, Paula Hino

**Affiliations:** I Universidade Federal de São Paulo. São Paulo, São Paulo, Brazil; II Secretaria Municipal de Saúde de São Paulo. São Paulo, São Paulo, Brazil; III Universidade Federal de Goiás. Goiânia, Goiás, Brazil

**Keywords:** Tuberculosis, Primary Health Care, Health Services Evaluation, Coronavirus Infections, Public Health., Tuberculosis, Atención Primaria de Salud, Evaluación de los Servicios de Salud, Infecciones por Coronavirus, Salud Pública.

## Abstract

**Objectives:**

to investigate which tuberculosis monitoring actions were modified in Primary Health Care services in the eastern region of the city of São Paulo.

**Methods:**

a cross-sectional study based on primary and secondary data. Key informants from 20 health units were interviewed using a semi-structured questionnaire. Descriptive and analytical statistics were used for data analysis.

**Results:**

in 2020, 2021 and 2022, there was an increase in working hours and a significant change in the work process. Changes in tuberculosis detection actions were identified, with an association between the change in consultation offer and the year 2020 when compared to 2022 (p=0.02). A decrease in the tuberculosis incidence rate and an increase in the mortality rate were observed between the years.

**Conclusions:**

the repercussions of the pandemic on tuberculosis detection measures are highlighted, which had an impact on the epidemiological data of the disease in the municipality.

## RESUMEN

**Objetivos:**

investigar qué acciones de seguimiento de la tuberculosis fueron modificadas en los servicios de Atención Primaria de Salud de la región este de la ciudad de São Paulo.

**Métodos:**

estudio transversal basado en datos primarios y secundarios. Se entrevistó a informantes clave de 20 unidades de salud mediante un cuestionario semiestructurado. Se utilizó estadística descriptiva y analítica para analizar los datos.

**Resultados:**

en 2020, 2021 y 2022 se produjo un aumento de la jornada laboral y un cambio importante en el proceso de trabajo. Se identificaron cambios en las acciones de detección de tuberculosis, existiendo asociación entre el cambio en la oferta de consultas y el año 2020, en comparación con 2022 (p=0,02). Se observó una disminución en la tasa de incidencia de tuberculosis y un aumento en la tasa de mortalidad entre los años.

**Conclusiones:**

se destacan las repercusiones de la pandemia en las medidas de detección de tuberculosis, las cuales repercutieron en los datos epidemiológicos de la enfermedad en el municipio.

## INTRODUCTION

According to the World Health Organization (WHO), tuberculosis (TB) is an infectious disease that causes concern to health authorities because every year, on average, 10 million people fall ill and 1.5 million of them die due to TB, even though there are treatment and means of prevention for TB^(1)^.

TB remains a challenge, as it remains one of the leading causes of death from infectious diseases worldwide. Therefore, the Sustainable Development Goals (SDGs) stand out, addressing global issues, including TB, which is included in the third goal (Health and Well-being) and has the goal of ending its epidemic by 2030^(2)^.

TB is socially determined, which makes certain populations more susceptible to developing the disease. It is important to recognize that social inequalities must be addressed through public policies, since, like TB, these disparities cause vulnerability and therefore require attention in the political-social sphere^(3)^.

Therefore, Primary Health Care (PHC) plays a relevant role in TB diagnosis and treatment, with actions, such as detecting cases through the active search for respiratory symptoms, which contribute in an essential way to prevention, integrating one of the investigative pillars established by the National Plan to End TB as a public health problem^(4)^.

Furthermore, this TB elimination plan was created as a guideline for tackling the disease in Brazil, providing support to health managers so that they can establish effective strategies to control the disease, according to the needs of each scenario. It also recognizes the commitment to the global “End TB Strategy” proposed by the WHO, in which targets were established to reduce the incidence coefficient (IC) by 90% and reduce the number of deaths from TB by 95% by 2035^(4)^.

Based on this overview, and with the official declaration by the WHO of a pandemic due to the new coronavirus in March 2020^(5)^ given the presentation of COVID-19 with symptoms very similar to those of TB, the obstacles to achieving the National Plan to End TB goals in Brazil were accentuated, with a 25% drop in diagnoses and a 26% increase in mortality at the end of that year^(6)^.

Furthermore, the 2021 Global TB Report highlights the negative impact of COVID-19 on the provision of essential services for TB care and reduction of its burden. The most profound problem resulting from COVID-19 was the 18% decrease in TB notifications, a figure that is similar to that observed in 2012, i.e., the equivalent of a ten-year setback. Furthermore, it is believed that the lack of access to TB diagnosis and treatment resulting from the difficulties faced during the COVID-19 pandemic has increased the number of deaths from the disease^(7)^.

A scoping review presented evidence about the impact of the COVID-19 pandemic on TB control since its onset, with the necessary social distancing measures that reflected in early detection and adherence to TB treatment. Moreover, the need to reorganize health services to meet the demands imposed by the pandemic and the deterioration of people’s living and working conditions worsened the context of social vulnerability with repercussions on TB incidence and implications for its control actions^(8)^.

Furthermore, a study that aimed to analyze the knowledge and practices of contacts of TB patients and health professionals in relation to risks to public health resulting from the interruption of pulmonary TB treatment showed that the interruption of pulmonary TB treatment is highly costly and fraught with stigma in the collective sense, due to the perpetuation of its transmission chain, which explains important negative consequences in the context of collective health^(9)^.

The pandemic has had an impact on TB control, given that, for instance, one of the most important measures to restrict COVID-19 virus circulation, social isolation, has made early diagnosis difficult through active screening for respiratory symptoms. In terms of organization of health services, it was necessary to rearrange them to meet the emerging demands of COVID-19, which had an impact on the structure of care for TB patients, causing concern regarding compliance with the goals established by the “End TB Strategy”^(8)^.

Due to the scenario presented, given the reorganization of TB control actions, with a reduction in case notification and an increase in the number of deaths, there was a need to understand the changes in TB detection actions between 2020 and 2022.

## OBJECTIVES

To investigate which TB monitoring actions were modified in PHC services in the eastern region of the city of São Paulo.

## METHODS

### Ethical aspects

In compliance with the ethical principles surrounding research involving human beings, in accordance with Resolution 466/2012 of the Brazilian National Health Council, the study was approved by the *Universidade Federal de Goiás* Research Ethics Committee and the Municipal Health Department of São Paulo. Participants’ consent was recorded by signing the Informed Consent Form, which was presented before the interviews began.

### Study design, period and location

This is a cross-sectional study with a quantitative approach, guided by STrengthening the Reporting of OBservational studies in Epidemiology (STROBE) recommendations. It is part of a multicenter study called “Repercussion of the COVID-19 pandemic on TB in Brazilian capitals: reality and new perspectives in Primary Care”.

The research was carried out in the eastern region of the city of São Paulo, with primary and secondary data on TB detection actions developed between 2020 and 2022 in PHC services. This region was chosen because it had the highest IC of TB in the city of São Paulo in the pre-pandemic period, with 64.6 cases/100 thousand inhabitants^(10)^. Furthermore, with an estimated population of over 2.5 million inhabitants, the eastern region has 76.8% coverage of PHC and 51.9% of Family Health Strategy^(11)^.

### Sample; inclusion and exclusion criteria

The study population consisted of all health units that were part of PHC of the city of São Paulo (n=117). A sample calculation was performed, considering the estimated proportion of 50% outcome and 10% absolute error value of the estimate, resulting in a total of 20 health units. These were selected through simple random sampling, using the RStudio software with the sample function (N, n, replace = FALSE).

For the interviews, a key informant was defined as a health professional who worked directly in TB control, indicated by the manager or the unit team. The inclusion criterion was a professional who had been active for at least three months in the PHC service since before the pandemic period. Health units that did not have TB cases during the pandemic constituted the exclusion criterion.

### Study protocol

Data collection was carried out by a team of researchers from December 2022 to March 2023, with this group receiving specific training to conduct data collection.

Concerning primary data, variables were selected from the instrument used in the multicenter project, with questions that addressed PHC unit characteristics in general and focused on TB care and health actions to detect TB cases. Furthermore, these had multiple-choice, dichotomous (yes-no) and Likert scale (0-10) response patterns. The instrument was built on the Research Electronic Data Capture (REDCap) platform and used by interviewers during data collection.

As for secondary data, epidemiological and operational indicators for TB from 2019 to 2022 were made available by the Health Surveillance Coordination Office of the city of São Paulo.

### Analysis of results, and statistics

Primary data were extracted from REDCap and entered into an Excel^®^ spreadsheet. Based on a study that assessed PHC performance^(12)^, Likert scale variables were categorized as: 0, which expresses “no change”; 1 to 3, “little change”; 4 to 7, “moderate change”; and 8 to 10, “great change”. A descriptive analysis was performed regarding unit characterization, actions related to TB care and detection actions, reporting the absolute and relative frequencies of categorical variables. PHC unit characterization was defined as independent variable, and variables of health actions related to the detection of TB cases were defined as outcome variables.

The association hypotheses were assessed using Generalized Mixed Models (GMM), with Gaussian distribution and linear link function. According to the significance presented in GMM, the Bonferroni post-hoc test was used to compare the mean changes in detection actions (on a Likert scale) for the years (2020, 2021, and 2022) and type of unit (family health and traditional basic health). A statistical significance level of less than 5% (p < 0.05) was adopted, using the statistical programs Jamovi version 2.3.28 and RStudio version 4.1.3 to perform the analyses.

## RESULTS

Study sample consisted of 20 health units, the majority of which were Family Health Strategy units (65%) and had the highest absence of nursing assistants/technicians (80%) and physician (65%) (Table 1).

**Table 1:** Characteristics of health units and tuberculosis control actions in Primary Health Care in the eastern health region, São Paulo, São Paulo, Brazil, 2020-2022

Variable	Yes	No
	n(%)	n(%)
Type of health unit		
Family health unit	13(65)	7(35)
Traditional basic unit	7(35)	13(65)
Professionals on leave or relocated (due to training)		
Nursing technician/assistant	16(80)	4(20)
Physician	13(65)	7(35)
Nurse	11(55)	9(45)
Community health worker	11(55)	9(45)
Increase in working hours (year)		
2020	17(85)	3(15)
2021	18(90)	2(10)
2022	12(60)	8(40)
Change in physical space organization (year)		
2020	19(95)	1(5)
2021	19(95)	1(5)
2022	18(90)	2(10)
Change in work process (year)		
2020	20(100)	0
2021	20(100)	0
2022	18(90)	2(10)
Offer of continuing education activities on tuberculosis (year)		
2020	11(55)	9(45)
2021	11(55)	9(45)
2022	17(85)	3(15)
Professional responsible for tuberculosis control actions in the unit (by training)		
Nurse	20(100)	0
Nursing technician/assistant	16(80)	4(20)
Physician	12(60)	8(40)
Community health worker	7(35)	13(65)
Telemonitoring offer		
For the care of people with tuberculosis	7(35)	13(65)

In relation to unit characteristics, the majority reported changes in working hours (85%, 90% and 60%), in physical space organization (95%, 95% and 90%) and in the work process (100%, 100% and 90%) during the pandemic period (2020 to 2022).

Concerning TB care, all units reported having a nurse as the professional responsible for TB control actions, together with a nursing technician/assistant (80%), followed by a physician (60%). A minority of the sample claimed to offer telemonitoring (35%), with daily consultations for people with signs and symptoms of TB.

Concerning control actions, 2020 was the year that presented the most changes, with 40% in the offer of consultations for respiratory symptoms, 25% in the request for sputum tests and chest X-rays and 15% in the notification of TB cases (Table 2).

**Table 2:** Changes in tuberculosis control actions in Primary Health Care in the eastern health region, São Paulo, São Paulo, Brazil, 2020-2022

Variable	No change	Low	Moderate	High
	n(%)	n(%)	n(%)	n(%)
Consultation offer for respiratory symptoms (year)				
2020	12(60)	1(5)	4(20)	3(15)
2021	12(60)	1(5)	4(20)	3(15)
2022	16(80)	2(10)	2(10)	0
Request for sputum examination for diagnosis and control (year)				
2020	15(75)	0	4(20)	1(5)
2021	16(80)	0	2(10)	2(10)
2022	18(90)	0	2(10)	0
Request for chest X-ray for diagnosis (year)				
2020	15(75)	0	5(25)	0
2021	15(75)	0	4(20)	1(5)
2022	19(95)	0	1(5)	0
Notification of tuberculosis cases (year)				
2020	17(85)	0	2(10)	1(5)
2021	18(90)	0	2(10)	0
2022	19(95)	1(5)	0	0

When assessing changes in detection actions, it was observed that the year variable showed a significant association with the change in the offer of consultations for respiratory symptoms (F_2;38_= 4.601; p=0.016), i.e., the pandemic period influenced the offer of consultations for patients with respiratory symptoms of TB (Table 3), for which the year 2022 presented a lower change value, on average, when compared to the year 2020 (Table 4).

**Table 3:** Association of changes in tuberculosis detection actions with the pandemic period and type of unit in the eastern health region, São Paulo, São Paulo, Brazil, 2020-2022

Variable	Year		Type of unit	
	F (gl)	*p* value	F (gl)	*p* value
Offer of consultation for respiratory symptoms	4.601 (2;38)	0.016^*^	0.406 (1;18)	0.532
Request for sputum examination for diagnosis	1.737 (2;38)	0.19	0.001 (1;18)	0.981
Request for chest x-ray for diagnosis	2.89 (2;38)	0.068	0.665 (1;18)	0.425
Notification of tuberculosis cases	2.74 (2;38)	0.077	2.803 (1;18)	0.111

* *Statistical significance (p < 0.05).*

**Table 4:** Average difference between the change in tuberculosis detection action during the pandemic period in the eastern health region, São Paulo, São Paulo, Brazil, 2020-2022

Variable	Comparisons	Mean difference	Standard error	*p* value^*^
	**Year**			
Offer of consultation for respiratory symptoms^**^	2020 - 2021	0.15	0.658	1
	2020 - 2022	1.80	0.658	0.028
	2021 - 2022	1.65	0.658	0.05

* Bonferroni post-hoc test;

** Variable on Likert scale (0-10).

In relation to epidemiological data, a decrease in CI was observed in 2020 (54.36 cases/100 thousand inhabitants), 2021 (53.73 cases/100 thousand inhabitants) and 2022 (59.14 cases/100 thousand inhabitants), when compared to 2019 (64.23 cases/100 thousand inhabitants).

Concerning the mortality rate, the opposite was found: the years 2019 (3.37 deaths/100,000 inhabitants) and 2020 (3.15 deaths/100,000 inhabitants) presented lower proportions, when compared to the years 2021 (3.89 deaths/100,000 inhabitants) and 2022 (4.38 deaths/100,000 inhabitants).

As for the operational data, it was identified that the pre-pandemic period presented a lower proportion of respiratory symptomatic patients examined, with 64.27%, compared to 85.13% (2020), 90.04% (2021) and 90.98% (2022) of people with signs of TB symptoms examined during the pandemic.

## DISCUSSION

The study aimed to identify TB detection actions that underwent changes during the COVID-19 pandemic within PHC in the eastern region of the city of São Paulo, in which changes were observed in relation to its organization and structure.

Increased working hours were reported during the pandemic. In Brazil, primary health supports the reduction of social inequalities due to its central role in the health network, thus being the user’s gateway to care, disease prevention, health education, among other activities^(13)^. However, the new demand imposed by COVID-19, together with the overcrowding of health units, justifies the overload suffered by PHC. In addition to this, it did not have the technical support to provide the necessary care for this COVID-19 demand, since patients were characterized as medium to high complexity and the primary service does not fit this user profile.

Furthermore, referrals to more complex services became more difficult and time-consuming, due to the total occupancy of beds in highly complex hospitals, which meant that patients remained in a service where their needs would not be met as well as having a more unfavorable prognosis during their stay in a hospital with overcrowded beds^(14)^.

A study carried out in the city of Rio de Janeiro, which aimed to recommend possibilities for the government in Brazil to combat COVID-19, through the Brazilian Health System, presented reports from professionals who faced overcrowding in PHC which, like secondary and tertiary health services, also suffered from the increase in the number of users, due to care network disarticulation for patients with COVID-19^(15)^.

This fact corroborates the findings that showed an increase in working hours and changes in the work process, given the need for health professionals to remain in the units, meeting the existing demand and adding to the new demand from users, as well as the need to compensate for the lack of professionals, due to absences due to comorbidity or illness^(16)^.

Most of health services in this study reported changes in physical space, justified by recommendations to reorganize health units in order to optimize them to separately serve the new flow of respiratory symptomatic patients^(17)^. This change generated significant changes in the PHC work process, which involved both structural changes and changes in the services offered^(18)^.

In 2020, a Basic Health Unit (BHU) in southern Bahia used a service organization strategy to deal with the COVID-19 pandemic, in which ongoing employee education was one of the pillars that supported it. These activities were implemented by the BHU itself before the COVID-19 emergency, without relying on health authorities to pass them on, which was essential because these actions were reduced during the pandemic. As a result, these activities supported the team’s coping strategies and a better community response to restriction measures and respiratory etiquette, which resulted in the region covered by the BHU being the one with the fewest confirmed cases of acute respiratory syndrome^(19)^.

In contrast to this reality, a reduction in the supply of continuing education activities for TB was reported at the height of the pandemic period, which may also have had consequences for professionals in the differential diagnosis of TB, since the focus was on COVID-19, hindering the detection of new cases and compromising the health team’s coping strategies.

Due to social isolation and the need for lockdown to prevent the spread of the COVID-19 virus, active search activities were compromised, which forced services to promote an alternative to continue this program. The use of telemedicine was indicated by part of the study sample, and it was shown to be a positive tool in this context, such as its application in monitoring the intake of TB medications in health facilities that used it. However, it has limited access to devices with an internet connection to hold meetings with health professionals.

Furthermore, a cross-sectional study conducted in Florianópolis highlighted that, compared to in-person consultations carried out by the same professional, teleconsultations were characterized by shorter consultation times and fewer referrals. This may indicate a limitation in the clinical analysis of patients through this means, which would lead to late or erroneous diagnoses and an unfavorable prognosis^(20)^.

Due to the focus of professionals being on the new acute respiratory syndrome, a change in the TB detection action was observed, due to the report of an increase in the time between the identification of the disease and the submission of the respective mandatory notification form. It can be inferred that the postponement of notification may lead to the recording of erroneous data in the official surveillance agencies, making it difficult to identify the epidemiological reality of the region in question^(21)^.

A health unit reported an increase in the number of TB case notifications, based on the perspective of similar symptoms of both diseases, increasing surveillance to make the correct diagnosis. Research conducted in Ghana^(22)^, in 2021, corroborated this finding, as it highlighted the importance of practicing the differential diagnosis between COVID-19 and TB in the period, due to the similar symptoms of these diseases, which contributed to identification and adequate follow-up of treatment. It can be deduced that this activity contributes both to the notification of new cases and to referral for appropriate treatment, reducing mortality from TB^(23)^.

Although operational indicators indicate an increase in the proportion of respiratory symptomatic patients examined in 2020, 2021 and 2022, compared to what was observed in 2019, the data published by the WHO Global TB Report 2023^(24)^ and the epidemiological indicators of the eastern region show numbers that indicate a reduction in the notification of cases of this disease during the pandemic period, following the annual trend, compared to the pre-pandemic period.

This data is reinforced when analyzing that changes resulting from the work process reorganization in PHC also encompassed the care offered to patients with respiratory symptoms^(20)^, a fundamental detection strategy to enable adequate diagnosis and treatment of patients. According to an analysis of the Brazilian health system^(25)^, which estimated its functionality and resilience, it indicated that there was a reduction in the supply of vacancies for health service workers, as well as the services offered by this network. This supports the findings of this study, which showed a change in the availability of this service in the years 2020 and 2021, when compared to the year 2022, whose informants reported smaller changes, in addition to this being a year in which services were returning to pre-pandemic configurations.

As a consequence of this, it is likely that people have been infected with Koch’s bacillus during the COVID-19 pandemic; however, diagnosis and treatment have been postponed due to disruptions caused by the pandemic in the health system, affecting access to health services, in addition to the fear of being exposed to other diseases when attending the BHU.

### Study limitations

Understanding the importance of this study, the limitation is that the collection scenario was only one health region among the six present in the city of São Paulo, which restricts the generalization of the findings.

### Contributions to health, nursing, or public policy

The results found allow for a situational diagnosis of the scenario and, in this way, provide support for strengthening the TB control program and health professionals’ actions, aiming at eliminating the disease as a public health problem.

## CONCLUSIONS

The COVID-19 pandemic has had an impact on TB detection efforts, particularly in relation to the provision of consultations for patients with respiratory symptoms, in which there has been an increase in the provision of remote care and in the reporting of these cases, which, in part, have suffered an increase in the time taken to carry out this action. Changing the factors that make up the strategy for tracking new TB cases may disrupt the program for controlling this disease and influence the epidemiological figures for the region it covers.

It is essential to carry out new studies in order to monitor the trend in the reporting of new cases of respiratory symptoms, which provides continuity to the analysis initiated in this study, which will see whether the disruptions that occurred during the pandemic will spread to future control actions. The discontinuation of these activities may further compromise the control goals of the “End TB Strategy”, which may deepen unfavorable prognosis indicators and intensify the vulnerability of the groups most susceptible to TB.

In the context of TB, it is believed that the SDGs have potential in tackling the disease through an integrated approach (response capacity through broad public policies that consider sustainable development, addressing its structural and socioeconomic causes), multisectoral collaboration (partnerships between governments, international organizations, the private sector and civil society), inclusion and equity actions, especially for vulnerable populations, and incentives for research, among others. With this, the health system can be strengthened and will be reflected in the achievement of other objectives, providing a healthier and more sustainable future.

## Data Availability

The research data are available only upon request.
