# Supplementary material for: Tuberculosis detection actions in Primary Health Care during the COVID-19 pandemic
Source: Rev Bras Enferm. 2025 Dec 8;78(5):e20240389. doi: 10.1590/0034-7167-2024-0389 (PMC12707759; doi:10.1590/0034-7167-2024-0389)
Supplement: Translation

# Translation: Ações de detecção da tuberculose na Atenção Primária à Saúde durante a pandemia de COVID-19

**DOI:** 10.1590/0034-7167-2024-0389pt

**Authors:** Isabella Carlini Bulizani, Stephanie Ribeiro, Ana Carolina Silva, Thais Tiemi Yamamoto, Rachel Russo Leite, Hugo Fernandes, Roxana Isabel Cardozo Gonzales, Paula Hino

**Affiliations:** I Universidade Federal de São Paulo. São Paulo, São Paulo, Brasil; II Secretaria Municipal de Saúde de São Paulo. São Paulo, São Paulo, Brasil; III Universidade Federal de Goiás. Goiânia, Goiás, Brasil

**Keywords:** Tuberculose, Atenção Primária à, Saúde, Avaliação de Serviços de Saúde, Infecções por Coronavírus, Saúde Pública.

## Abstract

**Objetivos:**

investigar quais ações de monitoramento da tuberculose foram modificadas nos serviços de Atenção Primária à Saúde da região leste do município de São Paulo.

**Métodos:**

estudo transversal com base em dados primários e secundários. Foram entrevistados informantes-chave de 20 unidades de saúde com apoio de um questionário semiestruturado. Utilizaram-se estatísticas descritivas e analíticas para análise dos dados.

**Resultados:**

em 2020, 2021 e 2022, houve aumento da carga horária de trabalho e importante alteração no processo de trabalho. Foram identificadas alterações nas ações de detecção da tuberculose, com associação entre a alteração de oferta de consulta e o ano de 2020, quando comparado a 2022 (p=0,02). Observaram-se diminuição no coeficiente de incidência da tuberculose e aumento no coeficiente de mortalidade entre os anos.

**Conclusões:**

são destacadas as repercussões da pandemia nas medidas de detecção da tuberculose, as quais geraram impacto nos dados epidemiológicos da enfermidade no município.

## INTRODUÇÃO

De acordo com a Organização Mundial da Saúde (OMS), a tuberculose (TB) é uma doença infecciosa que causa preocupação às autoridades de saúde em razão de todo ano, em média, 10 milhões de pessoas adoecerem e 1,5 milhões morrerem devido à TB, ainda que exista tratamento e meios de prevenção para o adoecimento por TB^(1)^.

A TB persiste como desafio, visto que persiste como uma das principais causas de morte por doenças infecciosas no mundo. Sendo assim, destacam-se os Objetivos do Desenvolvimento Sustentável (ODS) que abordam questões globais, incluindo a TB, que está contemplada no terceiro objetivo (Saúde e Bem-estar) e apresenta como meta acabar com a sua epidemia até 2030^(2)^.

O adoecimento pela TB é socialmente determinado, o que torna certas populações mais suscetíveis ao desenvolvimento da doença. Destaca-se a necessidade do reconhecimento de que desigualdades sociais devem ser enfrentadas por meio de políticas públicas, pois, a exemplo da TB, essas disparidades são causadoras da vulnerabilidade e, portanto, necessitam de atenção no âmbito político-social^(3)^.

Sendo assim, a Atenção Primária à Saúde (APS) tem papel relevante no diagnóstico e tratamento da TB, com ações a exemplo da detecção de casos através da busca ativa de sintomáticos respiratórios, que contribuem de forma essencial na prevenção, integrando um dos pilares investigativos estabelecidos pelo Plano Nacional pelo Fim da TB como problema de saúde pública^(4)^.

Ademais, esse plano de eliminação da TB foi criado como uma orientação para o seu enfrentamento no Brasil, fornecendo subsídio aos gestores em saúde para que se estabeleçam estratégias efetivas de controle da doença, de acordo com a necessidade de cada cenário. Neste, também é reconhecido o compromisso com a estratégia global “Estratégia Fim da TB”, em inglês, *End TB Strategy*, proposta pela OMS, no qual foram estabelecidas metas de redução de 90% do coeficiente de incidência (CI) e redução de 95% no número de mortes até 2035 por TB^(4)^.

A partir desse panorama, e com a declaração oficial da OMS de pandemia pelo novo coronavírus em março de 2020^(5)^, devido à apresentação da COVID-19 com uma sintomatologia muito semelhante à da TB, foram acentuados os obstáculos para o alcance das metas do Plano Nacional pelo fim da TB no Brasil, verificando uma queda dos diagnósticos de 25% e aumento da mortalidade de 26% no final daquele ano^(6)^.

Além disso, o Relatório Global da TB de 2021 destaca o impacto negativo da COVID-19 na promoção de serviços essenciais na assistência à TB e na redução de sua carga. A problemática mais profunda decorrente da COVID-19 foi a diminuição de 18% nas notificações de TB, valor que se equipara ao observado em 2012, ou seja, o equivalente a um retrocesso de dez anos. Ademais, acredita-se que a dificuldade de falta de acesso ao diagnóstico e ao tratamento da TB decorrente das dificuldades enfrentadas durante a pandemia de COVID-19 tenha elevado o número de mortes pela doença^(7)^.

Revisão de escopo apresentou evidências acerca do impacto da pandemia de COVID-19 no controle da TB, desde seu início, com as necessárias medidas de distanciamento social que refletiram na detecção precoce e adesão ao tratamento da TB. Além disso, a necessidade de reorganização dos serviços de saúde para atender às demandas impostas pela pandemia e a deterioração das condições de vida e de trabalho das pessoas agravaram o contexto de vulnerabilidade social com repercussão na incidência da TB e implicações nas suas ações de controle^(8)^.

Outrossim, estudo que teve como objetivo analisar os saberes e práticas dos contatos dos portadores de TB e dos profissionais de saúde em relação aos riscos à saúde pública decorrentes da interrupção do tratamento da TB pulmonar expôs que a interrupção do tratamento da TB pulmonar é altamente custosa e carregada de estigma no sentido coletivo, pela perpetuação de sua cadeia de transmissão, o que explicita importantes consequências negativas no âmbito da saúde coletiva^(9)^.

A pandemia acarretou impactos no controle da TB, haja vista, por exemplo, que uma das medidas mais importantes para a restrição de circulação do vírus da COVID-19, o isolamento social, dificultou o diagnóstico precoce pela busca ativa de sintomáticos respiratórios. Já em relação à organização do serviço de saúde, foi necessário um rearranjo para as demandas emergentes da COVID-19, o qual reverberou na estrutura de assistência do paciente com TB, causando preocupação em relação ao cumprimento das metas estabelecidas pela “Estratégia Fim da TB”^(8)^.

Em razão do cenário exposto, diante da reorganização das ações de controle da TB, com redução da notificação de casos e aumento do número de mortes, é expressa a necessidade de compreender as mudanças nas ações de detecção da TB entre 2020 e 2022.

## OBJETIVOS

Investigar quais ações de monitoramento da TB foram modificadas nos serviços de APS da região leste do município de São Paulo.

## MÉTODOS

### Aspectos éticos

Respeitando os princípios éticos que envolvem as pesquisas com seres humanos, em concordância com a Resolução n^o^ 466/2012 do Conselho Nacional de Saúde, o estudo obteve aprovação do Comitê de Ética em Pesquisa da Universidade Federal de Goiás e da Secretaria Municipal de Saúde de São Paulo. A anuência dos participantes foi registrada pela assinatura do Termo de Consentimento Livre e Esclarecido, que foi apresentado antes de iniciar as entrevistas.

### Desenho, período e local do estudo

Trata-se de estudo transversal e de abordagem quantitativa, guiado pelas recomendações do *STrengthening the Reporting of OBservational studies in Epidemiology* (STROBE). Este integra estudo multicêntrico denominado “Repercussão da Pandemia da COVID-19 na TB em capitais brasileiras: realidade e novas perspectivas na Atenção Primária”.

A pesquisa foi realizada na região leste do município de São Paulo, com dados primários e secundários sobre as ações de detecção da TB desenvolvidas entre 2020 e 2022 nos serviços de APS. Optou-se pelo recorte dessa região por apresentar o maior CI de TB do município de São Paulo no período pré-pandêmico, com 64,6 casos/100 mil habitantes^(10)^. Além disso, com uma população estimada de mais de 2,5 milhões de habitantes, a região leste dispõe de 76,8% de cobertura de APS e de 51,9% da Estratégia Saúde da Família^(11)^.

### Amostra; critérios de inclusão e exclusão

A população do estudo foi composta por todas as unidades de saúde que integravam a APS do município de São Paulo (n=117). Foi realizado cálculo amostral, considerando a proporção estimada do desfecho de 50% e o valor do erro absoluto da estimativa de 10%, resultando em um quantitativo de 20 unidades de saúde. Estas foram selecionadas por meio de amostragem aleatória simples, utilizando o *software* RStudio com a função sample (N, n, *replace* = *FALSE*).

Para as entrevistas, definiu-se como informante-chave um profissional de saúde que atuava diretamente no controle da TB, indicado pelo gestor ou pela equipe da unidade. Como critério de inclusão, foi considerado o profissional que estava ativo há pelo menos três meses no serviço de APS desde antes do período pandêmico. Já unidades de saúde que não tiveram casos de TB durante a pandemia constituíram o critério de exclusão.

### Protocolo do estudo

A coleta de dados foi realizada por uma equipe de pesquisadores no período de dezembro de 2022 a março de 2023, com esse grupo recebendo treinamento específico para a condução da coleta de dados.

Quanto aos dados primários, as variáveis foram selecionadas do instrumento utilizado no projeto multicêntrico, com questões que abordavam as características das unidades de APS em geral e voltadas para a atenção à TB e ações de saúde para detecção de casos de TB. Ademais, essas tinham padrões de resposta do tipo múltipla escolha, dicotômica (sim-não) e escala Likert (0-10). O instrumento foi construído na plataforma *Research Electronic Data Capture* (REDCap) e utilizado pelos entrevistadores durante a coleta de dados.

Quanto aos dados secundários, foram disponibilizados pela Coordenadoria de Vigilância em Saúde do município de São Paulo indicadores epidemiológicos e operacionais da TB dos anos de 2019 a 2022.

### Análise dos resultados e estatística

Os dados primários foram extraídos do REDCap e alocados em planilha do Excel^®^. Com base em estudo que avaliou o desempenho da APS^(12)^, as variáveis da escala Likert foram categorizadas em: 0, que expressa “sem alteração”; de 1 a 3, “pouca alteração”; de 4 a 7, “alteração moderada”; e de 8 a 10, “grande alteração”. Realizou-se a análise descritiva referente à caracterização das unidades, das ações relacionadas à atenção a TB e das ações de detecção, reportando as frequências absolutas e relativas das variáveis categóricas. Foi definida como variável independente a caracterização das unidades de APS, e como variáveis de desfecho, as variáveis de ações de saúde relacionadas à detecção de casos de TB.

As hipóteses de associação foram avaliadas via *Generalized Mixed Models* (GMM), com distribuição gaussiana e função de ligação linear. De acordo com a significância apresentada no GMM para a comparação das médias de alteração das ações de detecção (em escala Likert) para os anos (2020, 2021 e 2022) e tipo de unidade (saúde da família e básica tradicional), utilizou-se o *post-hoc* de Bonferroni. Adotou-se o nível de significância estatística menor que 5% (p < 0,05), utilizando os programas estatísticos Jamovi, versão 2.3.28, e RStudio, versão 4.1.3, para realizar as análises.

## RESULTADOS

A amostra do estudo foi composta por 20 unidades de saúde, das quais, em sua maioria, eram Estratégia Saúde da Família (65%) e apresentaram maior afastamento de auxiliar/técnicos de enfermagem (80%) e médicos (65%) (Tabela 1).

**Tabela 1:** Características das unidades de saúde e das ações de controle da tuberculose na Atenção Primária à Saúde da região de saúde leste, São Paulo, São Paulo, Brasil, 2020-2022

Variável	Sim	Não
	n(%)	n(%)
Tipo de unidade de saúde		
Unidade saúde da família	13(65)	7(35)
Unidade básica tradicional	7(35)	13(65)
Profissionais afastados ou realocados (por formação)		
Técnico/auxiliar de enfermagem	16(80)	4(20)
Médico	13(65)	7(35)
Enfermeiro	11(55)	9(45)
Agente comunitário de saúde	11(55)	9(45)
Aumento na carga horária de trabalho (ano)		
2020	17(85)	3(15)
2021	18(90)	2(10)
2022	12(60)	8(40)
Mudança na organização do espaço físico (ano)		
2020	19(95)	1(5)
2021	19(95)	1(5)
2022	18(90)	2(10)
Mudança no processo de trabalho (ano)		
2020	20(100)	0
2021	20(100)	0
2022	18(90)	2(10)
Oferta de atividade de educação permanente sobre tuberculose (ano)		
2020	11(55)	9(45)
2021	11(55)	9(45)
2022	17(85)	3(15)
Profissional responsável pelas ações de controle da tuberculose na unidade (por formação)		
Enfermeiro	20(100)	0
Técnico/auxiliar de enfermagem	16(80)	4(20)
Médico	12(60)	8(40)
Agente comunitário de saúde	7(35)	13(65)
Oferta de telemonitoramento		
Para a atenção às pessoas com tuberculose	7(35)	13(65)

Em relação às características das unidades, foram relatadas pela maioria mudanças na carga horária (85%, 90% e 60%), na organização do espaço físico (95%, 95% e 90%) e no processo de trabalho (100%, 100% e 90%) durante o período da pandemia (2020 a 2022).

No que diz respeito à atenção à TB, todas as unidades referiram ter o enfermeiro como profissional responsável pelas ações de controle da TB, juntamente com o técnico/auxiliar de enfermagem (80%), seguido do médico (60%). A minoria da amostra alegou oferecer telemonitoramento (35%), com oferta de consulta diária, para pessoas com sinais e sintomas de TB.

Acerca das ações de controle, o ano de 2020 foi o que mais apresentou alterações, com 40% na oferta de consulta para sintomáticos respiratórios, 25% na solicitação do exame de escarro e de radiografia de tórax e 15% na notificação dos casos de TB (Tabela 2).

**Tabela 2:** Alteração das ações de controle da tuberculose na Atenção Primária à Saúde da região de saúde leste, São Paulo, São Paulo, Brasil, 2020-2022

Variável	Sem alteração	Baixa	Moderada	Alta
	n(%)	n(%)	n(%)	n(%)
Oferta de consulta para sintomático respiratório (ano)				
2020	12(60)	1(5)	4(20)	3(15)
2021	12(60)	1(5)	4(20)	3(15)
2022	16(80)	2(10)	2(10)	0
Solicitação do exame de escarro para diagnóstico e controle (ano)				
2020	15(75)	0	4(20)	1(5)
2021	16(80)	0	2(10)	2(10)
2022	18(90)	0	2(10)	0
Solicitação da radiografia de tórax para diagnóstico (ano)				
2020	15(75)	0	5(25)	0
2021	15(75)	0	4(20)	1(5)
2022	19(95)	0	1(5)	0
Notificação dos casos de tuberculose (ano)				
2020	17(85)	0	2(10)	1(5)
2021	18(90)	0	2(10)	0
2022	19(95)	1(5)	0	0

Ao avaliar as alterações nas ações de detecção, observou-se que a variável ano apresentou associação significativa com a alteração na oferta de consulta para sintomáticos respiratórios (F_2;38_= 4,601; p=0,016), ou seja, o período pandêmico influenciou a oferta de consulta para sintomáticos respiratórios de TB (Tabela 3), para o qual o ano de 2022 apresentou valor inferior de alteração, em média, quando comparado ao ano de 2020 (Tabela 4).

**Tabela 3:** Associação das alterações das ações de detecção da tuberculose com o período pandêmico e tipo de unidade, da região de saúde leste, São Paulo, São Paulo, Brasil, 2020-2022

Variável	Ano		Tipo de unidade	
	F (gl)	Valor de *p*	F (gl)	Valor de *p*
Oferta de consulta para sintomáticos respiratórios	4,601 (2;38)	0,016^*^	0,406 (1;18)	0,532
Solicitação do exame de escarro para diagnóstico	1,737 (2;38)	0,19	0,001 (1;18)	0,981
Solicitação da radiografia de tórax para diagnóstico	2,89 (2;38)	0,068	0,665 (1;18)	0,425
Notificação dos casos de tuberculose	2,74 (2;38)	0,077	2,803 (1;18)	0,111

* *Significância estatística (p < 0,05).*

**Tabela 4:** Média da diferença entre a alteração da ação de detecção da tuberculose com o período pandêmico da região de saúde leste, São Paulo, São Paulo, Brasil, 2020-2022

Variável	Comparações	Média da diferença	Erro padrão	Valor de *p* ^*^
	**Ano**			
Oferta de consulta de sintomáticos respiratórios^**^	2020 - 2021	0,15	0.658	1
	2020 - 2022	1,80	0.658	0,028
	2021 - 2022	1,65	0.658	0,05

* Teste de post-hoc de Bonferroni;

** Variável em escala Likert (0-10).

Em relação aos dados epidemiológicos, observou-se diminuição do CI nos anos de 2020 (54,36 casos/100 mil habitantes), 2021 (53,73 casos/100 mil habitantes) e 2022 (59,14 casos/100 mil habitantes), quando comparado ao ano de 2019 (64,23 casos/100 mil habitantes).

No que diz respeito ao coeficiente de mortalidade, foi verificado o oposto: os anos de 2019 (3,37 óbitos/100 mil habitantes) e 2020 (3,15 óbitos/100 mil habitantes) apresentaram menores proporções, quando comparados aos anos de 2021 (3,89 óbitos/100 mil habitantes) e 2022 (4,38 óbitos/100 mil habitantes).

Quanto aos dados operacionais, foi identificado que o período pré-pandemia apresentou menor proporção de sintomáticos respiratórios examinados, com 64,27%, diante dos 85,13% (2020), 90,04% (2021) e 90,98% (2022) de pessoas com sinais de sintomas de TB examinadas durante a pandemia.

## DISCUSSÃO

O trabalho objetivou identificar as ações de detecção da TB que sofreram modificações no período da pandemia de COVID-19 no âmbito da APS da região leste do município de São Paulo, no qual foram observadas alterações em relação à sua organização e estrutura.

Foi relatada a ocorrência de aumento da carga horária de trabalho no período pandêmico. Na realidade brasileira, a APS apoia a redução das desigualdades sociais em razão de sua atuação central na Rede de Atenção à Saúde, sendo, assim, a porta de entrada do usuário à assistência, prevenção de doenças, educação em saúde, entre outras atividades^(13)^. Contudo, a nova demanda imposta pela COVID-19 junto à superlotação das unidades de saúde justifica a sobrecarga sofrida pela APS. Além disso, essa não possuía suporte técnico para proporcionar o atendimento necessário para essa demanda da COVID-19, já que os pacientes se caracterizavam como média a alta complexidade e o serviço primário não configura esse perfil de usuário.

Ademais, os encaminhamentos para os serviços de maior complexidade se tornaram mais difíceis e demorados, devido à ocupação total dos leitos nos hospitais de alta complexidade, o que implicava a permanência do paciente no serviço em que suas necessidades não seriam supridas, como também tenha um prognóstico mais desfavorável na sua permanência em um hospital com superlotação de leitos^(14)^.

Estudo desenvolvido no município do Rio de Janeiro, que teve como objetivo recomendar possibilidades de enfrentamento da COVID-19 pelo poder público no Brasil, através do Sistema Único de Saúde, apresentou relatos de profissionais que enfrentaram superlotação da APS que, tal como os serviços secundários e terciários da saúde, também sofreu com o aumento do número de usuários, em decorrência da desarticulação da rede de atenção ao paciente com COVID-19^(15)^.

Esse fato corrobora os achados que denotaram aumento da carga horária e alteração do processo de trabalho, haja vista a necessidade dos profissionais da saúde permanecerem nas unidades, suprindo a demanda já existente e somando a nova demanda dos usuários, como também pela necessidade de compensar a falta de profissionais, em decorrência de afastamentos por comorbidade ou adoecimento^(16)^.

A maioria dos serviços de saúde deste estudo relatou alterações no espaço físico, justificadas por recomendação de reorganização das unidades de saúde, a fim de otimizá-la para atender separadamente o novo fluxo de sintomáticos respiratórios^(17)^. Essa modificação gerou alterações significativas no processo de trabalho da APS, as quais envolveram tanto mudanças estruturais quanto dos serviços ofertados^(18)^.

Em 2020, uma Unidade Básica de Saúde (UBS) no sul da Bahia utilizou uma estratégia de organização do serviço para o enfrentamento da pandemia de COVID-19, em que a educação permanente dos funcionários se apresentava como um dos pilares que a sustentava. Essas atividades foram postas pela própria UBS em período anterior à emergência da COVID-19, sem depender de instâncias de saúde a repassarem, o que foi fundamental em virtude dessas ações terem sido reduzidas no período pandêmico. Como resultado, essas atividades subsidiaram estratégias de enfrentamento da equipe e melhor resposta da comunidade diante das medidas de restrição e etiqueta respiratória, o que fez com que a região abrangida pela UBS se colocasse como a com menos casos confirmados da síndrome respiratória aguda^(19)^.

Contrastando com essa realidade, foi reportada uma redução da oferta das atividades de educação permanente para TB no auge do período pandêmico, o que também pode ter trazido consequências para os profissionais no diagnóstico diferencial da TB, já que o foco estava voltado para a COVID-19, prejudicando a detecção de novos casos e comprometendo as estratégias de enfrentamento da equipe de saúde.

Em razão do isolamento social e necessidade de *lockdown* para evitar a disseminação do vírus da COVID-19, as atividades de busca ativa foram comprometidas, o que impôs aos serviços a necessidade de promover uma alternativa para a continuidade desse programa.

O uso da telemedicina foi indicado por parte da amostra do estudo, apresentando-se como uma ferramenta positiva nesse contexto, a exemplo da aplicação na observação da ingesta das medicações contra TB nos estabelecimentos de saúde que usufruíram dela. Contudo, possui a limitação do acesso a dispositivos com conexão de internet para serem realizados os encontros com o profissional de saúde.

Ademais, estudo transversal conduzido em Florianópolis destacou que, em comparação a consultas presenciais realizadas pelo mesmo profissional, as teleconsultas se caracterizaram por menor tempo de atendimento e menos encaminhamentos. Isso pode indicar uma limitação da análise clínica do paciente através desse meio, o que conduziria a diagnósticos tardios ou errôneos e um prognóstico desfavorável^(20)^.

Em decorrência do foco dos profissionais estar voltado para a nova síndrome respiratória aguda, foi observada a ocorrência de uma alteração na ação de detecção da TB, devido ao relato de aumento do tempo entre a identificação da doença e envio da respectiva ficha de notificação compulsória. Pode-se inferir que a postergação da notificação pode levar ao registro de dados errôneos nos órgãos oficiais de vigilância, dificultando a identificação da realidade epidemiológica da região em questão^(21)^.

Foi reportada por uma unidade de saúde a elevação do número de notificações de casos de TB, embasados na perspectiva da sintomatologia semelhante de ambas doenças, aumentando-se a vigilância para efetuar o correto diagnóstico. Pesquisa conduzida em Gana^(22)^, no ano de 2021, corroborou esse achado, pois destacou-se a importância da prática do diagnóstico diferencial entre COVID-19 e TB no período, em virtude da sintomatologia de essas doenças serem semelhantes, o que contribuiu para identificação e seguimento adequado ao tratamento. Deduz-se que essa atividade colabora tanto para a notificação de novos casos quanto para o encaminhamento para tratamento adequado, reduzindo a mortalidade para TB^(23)^.

Apesar de os indicadores operacionais apontarem o aumento da proporção de sintomáticos respiratórios examinados em 2020, 2021 e 2022, diante do observado em 2019, os dados publicados pelo Relatório Global de TB 2023 da OMS^(24)^ e os indicadores epidemiológicos do região leste trazem números que indicam a redução da notificação de casos dessa doença durante o período pandêmico, seguindo a tendência anual, comparados com o do período pré-pandêmico.

Esse dado é reforçado ao se analisar que mudanças advindas da reorganização do processo de trabalho na APS também englobaram o atendimento ofertado a sintomáticos respiratórios^(20)^, estratégia de detecção fundamental para viabilizar diagnóstico e tratamento adequados do paciente. De acordo com análise feita do sistema de saúde brasileiro^(25)^, que estimava sua funcionalidade e resiliência, indicou que houve redução de oferta de vagas para trabalhadores de serviços da saúde, como também os serviços oferecidos por essa rede. Isso corrobora os achados do presente estudo, o qual evidenciou uma alteração da disponibilização desse serviço nos anos de 2020 e 2021, quando comparados ao ano de 2022, cujos informantes relataram haver menores alterações, além desse se configurar como um ano em que os serviços estavam retomando as configurações pré-pandêmicas.

Como consequência disso, é provável que pessoas tenham se infectado pelo bacilo de Koch durante a pandemia de COVID-19, entretanto o diagnóstico e o tratamento foram postergados, em virtude das rupturas causadas pela pandemia no sistema de saúde, afetando o acesso aos serviços de saúde, acrescido do receio de se exporem a outras doenças ao comparecer na UBS.

### Limitações do estudo

Compreendendo a importância do estudo, destaca-se como limitação o cenário de coleta ser apenas uma região de saúde entre as seis presentes no município de São Paulo, o que restringe a generalização dos achados.

### Contribuições para as áreas da saúde, enfermagem, ou políticas públicas

Os resultados encontrados possibilitam a realização de um diagnóstico situacional do cenário e, dessa forma, fornecem subsídios para o fortalecimento do programa de controle da TB e das ações dos profissionais de saúde, visando à eliminação da doença como problema de saúde pública.

## CONCLUSÕES

A pandemia de COVID-19 repercutiu nas ações de detecção da TB, principalmente em relação à oferta de consulta para sintomáticos respiratórios, na qual se observou um crescimento da oferta de atendimentos remotos e na notificação desses casos, os quais, em parte, sofreram aumento de tempo para realizar essa ação. A mudança de fatores componentes da estratégia de rastreio de novos casos de TB pode desestruturar o programa de controle dessa doença e influenciar os números epidemiológicos da região que abrange.

É imprescindível a realização de novos estudos, a fim de acompanhar a tendência na notificação de casos novos de sintomáticos respiratórios, o que dá continuidade à análise iniciada no presente estudo, que verá se as rupturas que ocorreram na pandemia irão se propagar futuramente nas ações de controle. A descontinuidade dessas atividades pode comprometer ainda mais as metas de controle da estratégia “Estratégia Fim da TB”, podendo aprofundar indicadores de prognóstico desfavorável e intensificar a vulnerabilidade dos grupos mais suscetíveis à TB.

No cenário da TB, acredita-se que os ODS apresentam potencialidades no enfrentamento da doença, por meio de enfoque integrado (capacidade de resposta por meio de políticas públicas amplas que considerem o desenvolvimento sustentável, abordando suas causas estruturais e socioeconômicas), colaboração multissetorial (parceria entre governos, organizações internacionais, setor privado e sociedade civil), ações de inclusão e equidade, principalmente de populações vulneráveis, incentivo à pesquisa, entre outros. Com isto, o sistema de saúde poderá ser fortalecido e refletirá no alcance de outros objetivos, proporcionando um futuro mais saudável e sustentável.

## Data Availability

Os dados de pesquisa só estão disponíveis mediante solicitação.
